# The free-energy cost of interaction between DNA loops

**DOI:** 10.1038/s41598-017-12765-x

**Published:** 2017-10-03

**Authors:** Lifang Huang, Peijiang Liu, Zhanjiang Yuan, Tianshou Zhou, Jianshe Yu

**Affiliations:** 1 Research Centre of Applied Mathematics, Guangzhou University, Guangzhou, 510006 P.R. China; 2 School of Statistics and Mathematics, Guangdong University of Finance & Economics, Guangzhou, 510275 P.R. China; 3 Guangdong Province Key Laboratory of Computational Science, School of Mathematics and Computational Science, Sun Yat-Sen University, Guangzhou, 510275 P.R. China

## Abstract

From the viewpoint of thermodynamics, the formation of DNA loops and the interaction between them, which are all non-equilibrium processes, result in the change of free energy, affecting gene expression and further cell-to-cell variability as observed experimentally. However, how these processes dissipate free energy remains largely unclear. Here, by analyzing a mechanic model that maps three fundamental topologies of two interacting DNA loops into a 4-state model of gene transcription, we first show that a longer DNA loop needs more mean free energy consumption. Then, independent of the type of interacting two DNA loops (nested, side-by-side or alternating), the promotion between them always consumes less mean free energy whereas the suppression dissipates more mean free energy. More interestingly, we find that in contrast to the mechanism of direct looping between promoter and enhancer, the facilitated-tracking mechanism dissipates less mean free energy but enhances the mean mRNA expression, justifying the facilitated-tracking hypothesis, a long-standing debate in biology. Based on minimal energy principle, we thus speculate that organisms would utilize the mechanisms of loop-loop promotion and facilitated tracking to survive in complex environments. Our studies provide insights into the understanding of gene expression regulation mechanism from the view of energy consumption.

## Introduction

Transcription is a pivotal but also the most complex step during gene expression. Transcription of a gene is regulated often by promoter-proximal DNA elements and distal DNA elements that together determine the expression pattern of this gene. Enhancers activate promoters by directly contacting binding sites for transcription factors and chromatin-modifying enzymes via DNA looping^1–5^. Chromatin elements may form DNA loops of spatial structure in different manners, e.g., promoter-tethering elements in *Drosophila* that allow activation by specific enhancers over long distances are proposed to form DNA loops between sequences near the enhancer and the promoter^6,7^. Also for example, in bacteriophage λ, the CI protein forms a 2.3-kb DNA loop that brings a distal stimulatory site close to RNA polymerase at the PRM promoter^8^. Li, *et al*.^9^ thought that one major factor determining the formation of a DNA loop is the flexibility of chromatin, which may be modulated by histone acetylation and other modifications^10^. The specific interactions between chromatin elements can either assist enhancer–promoter looping by bringing the enhancer and the promoter closer together, or are thought to interfere with enhancer–promoter looping by placing them in separate loop domains^11^. In a more general sense, chromatin elements are linked in a highly complex network of DNA-looping interactions, where enhancers often (e.g., in eukaryotes) directly contact promoters over large genomic distances to regulate gene expression. Characterizing the principles underlying these distal enhancer-promoter contacts is fundamentally important for a full understanding of gene expression.

Revealing the mechanisms of how transcriptional enhancers control over 20,000 protein- coding genes to maintain cell-specific gene expression programs is a fundamental challenge in biology^12^. Studies^12–17^ suggested complex promoter structures formed by the interaction of DNA sites, especially by enhancer-gene interactions. These interactions are in essential biochemical, leading to stochastic transcription and further cell-to-cell variability^18,19^. In recent years, the study of chromosomal loop structures and loop-loop interactions has been receiving increasing attention. Priest, *et al*., used two well-characterized DNA-looping proteins^11^: Lac repressor and phage λ CI, to measure interactions between pairs of long DNA loops in *E. coli* cells in three possible topological patterns of two pairs of interacting sites on DNA, namely, side-by-side loops, nested loops, and alternating loops. They found that two DNA loops in the side-by-side structure do not affect each other; those in the nested structure assist each other’s formation consistent with the distance-shortening effect; and those in the alternating structure, where one looping element is placed within the other DNA loop, inhibit each other’s formation, thus providing clear support for the loop domain model for insulation. In addition, they argued that the combination of loop assistance and loop interference can provide strong specificity in long-range interactions. Another similar but important work is that Savitskaya, *et al*., experimentally observed^13^ that when a pair of repressor (Su and Hw) found in the gypsy retrotransposon and their binding sites are in between the enhancer and the promoter, the gene expression level is not decreased but increased. For this counter-intuitive phenomenon, Mirny, *et al*., conjectured that the Su and Hw pair shortens the distance between the enhancer and the promoter, leading to the rise of the expression level^14^. Despite these studies, it is still unclear to what extent this mechanism contributes to specific enhancer-gene interactions^12^ and which factors influence as well as how they affect the interaction between DNA loops. In this paper, we will address these questions from a viewpoint of energy dissipation. Our study is motivated by two important questions: The one is on the communication form between enhancer and promoter, namely, which of the direct looping and the facilitated–tracking looping has advantages^9,15,20^; The other is how DNA loops interact with one another, whether one DNA loop modulates the expression of another loop by changing the transcriptional rate^21–23^. Experiment data on the measured dependency of noise upon average expression can well fit the variations in transcriptional rates across chromosome^21^, but cannot specify the interaction between DNA loops and its effect on gene expression. This article tries to understand the interaction between DNA loops from the viewpoint of the minimal energy principle. For clarity, we will restrict our study to two representative interacting DNA loops (the one formed by silence genes, e.g., Su and Hw^13^, or Anchor and CTCF^12^ and the other by expressing genes, e.g., an enhancer and a promoter) that altogether may form the three fundamental structures as mentioned above.

As a fact, maximizing the energy available to cells for biosyntheses, growth, and division is essential for cell survival^24^, whereas the operation mechanisms of biological systems usually follow the minimal energy principle with^4–28^. For example, Wong, *et al*. showed that intron retention can regulate the activity of many genes, and this requires only a small amount of energy^29^, In addition, Wang, *et al*. showed that the path of differentiation in stem cell is obtained along the direction of minimum energy^30^. These works imply that using the minimal energy principle to infer biological mechanisms would be reasonable. In our case, the DNA loop formation is essentially a non-equilibrium process from the viewpoint of statistical thermodynamics, thus necessarily consuming energy. In fact, storage and retrieval of the genetic information in a cell are a dynamic process that requires the DNA to undergo structural rearrangements essential for transcriptional regulation in prokaryotic and eukaryotic cells^31^, DNA looping and the interaction between DNA loops are two prominent examples of such a structural deformation. This deformation belonging to energy-dependent chromatin remodeling necessarily dissipates energy. Interestingly, Chen, *et al*.^31^ showed that both loop association and loop dissociation at the DNA-repressor junctions depend on the elastic deformation of the DNA and protein, and that both looping and unlooping rates approximately scale with a looping factor that can well reflect the system’s deformation free energy. They found that the loop breakdown process at the DNA–protein interface is sensitive to the whole loop’s deformation, and that both looping and unlooping kinetics exhibit rather simple forms of scaling with the looping free energy. In addition, Coulon *et al*. ^32^ studied a general model of stochastic gene expression that can represent arbitrary prokaryotic or eukaryotic promoters, and revealed the potential activity of any promoter and its influence on gene expression from the perspective of energetic cost (e.g., they showed that the regime where molecular interactions underlying promoter dynamics have typical characteristics such as high mobility, functional redundancy and many alternate states/pathways results in direct and indirect energetic cost). The minimal energy principle would provide a good angle of view for understanding the regulatory mechanism of the interaction of DNA loops, but because of the complexity of biological regulations, energy calculation is nontrivial and needs to develop new methods.

We notice that the study of Chen, *et al*.^31^ and other similar works^33–35^ studied free energy dissipation but considered only the case of single DNA loops. Thus far, how the interaction between DNA loops consumes free energy and the relationship of the gene expression level with energy dissipation is not clear. In addition, classical thermodynamic models^36^ (used in the above mentioned works), which are based on the assumption that the level of gene expression is proportional to the equilibrium probability that RNA polymerase (RNAP) is bound to the promoter of interest, provide only a framework for understanding energy dissipation in gene expression, and in general cannot specify which important processes (e.g., the formation of DNA loops and their interaction) that can efficiently regulate gene expression levels contribute to energy consumption in the full system.

Here, we develop a new but intuitive computational approach to analyze the free energy cost of the interaction between chromatin loops. Specifically, we first map complex topological patterns for the interactions among DNA loops into a multistate model of stochastic transcription, where transition rates between promoter states are functions of the loop lengths (along DNA lines). Then, we propose a principle of free energy consumption (defined as the entropy production rate $\dot{W}=dW/dt$ in this paper, also called the dissipation rate of free energy), which decomposes this dissipation rate into two parts: the one for promoter dynamics (denoted by ${\dot{W}}_{p}$) and the other for promoter-mediated transcription dynamics (${\dot{W}}_{y}$). The former ${\dot{W}}_{p}$ is easily calculated using the standard statistical thermodynamics method^37–39^, while the latter ${\dot{W}}_{y}$ can be simply estimated from Fokker-Planck equations for the mRNA-concentration distributions with each derived by considering that the gene is at a single state (see the below Methods for details). Our energy dissipation model also can conveniently incorporate other factors such as the communication form between regulatory elements^12,13,16^. Importantly, apart from showing that a longer DNA loop needs more mean free energy consumption (defined as the ratio of the entropy production rate over the mean mRNA), we find that the promotion of two loops always reduces the mean free energy consumption whereas the suppression has the opposite effect, both independent of the topology of two DNA loops; in contrast to the mechanism of direct looping between regulatory elements, the facilitated-tracking mechanism dissipates less mean free energy and can enhance the mean mRNA expression, justifying the facilitated-tracking hypothesis in biology.

## Materials and Methods

### Hypotheses based on experimental evidence

**Figure 1:**
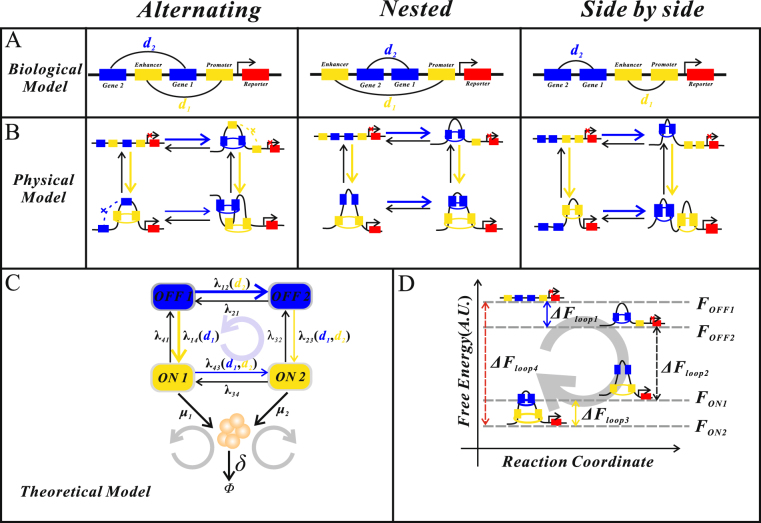
Schematic diagram for two interacting DNA loops. (**A**) Three fundamental biological structures, where two pairs: Gene1/Gene2 (yellow dock), enhancer/promoter (blue dock), may form two distinct topologies. (**B**) Physical structures for respective DNA–looping interactions in (**A**), which consider the possibility of looping. (**C**) Theoretical model by mapping the physical models into a 4-state model of gene expression, where transition rates between active and inactive states may depend the loop lengths along the DNA lines (called looping rates), and the grey loop with arrow represents free energy flow. (**D**) Free energy difference between unlooped and looped states.

In order to reveal the essential mechanism of how interacting DNA loops consumes free energy, here we consider only the interaction between two loops: the one formed by a pair of insulators: Su/Hw^13^ or Anchor/CTCF^12^, and the other formed by enhancer and promoter. For convenience, the former loop is denoted as the blue loop whereas the latter loop by the yellow loop, referring to Fig. 1A. According to ref.^11^, there are three possible connection topologies between these two pairs of DNA loops: cross-type structure (due to alternating loops), inline-type structure (due to nested loops), and independence-type structure (due to side-by-side loops).

Denote by *d*_1_ the length of the yellow loop along the DNA line, and by *d*_2_ the length of the blue loop also along the DNA line. Experimental evidence supports that alternating loops give loop interference, nested loops give loop assistance, and side-by-side loops do not interact^11,13^. We assume that the gene is expressed only after the yellow loop is formed. Note that in theory, a pair of DNA regulatory elements may form a loop but also may not form any loop, i.e., there are two possibilities. Thus, there are in total four possibilities for each of the three topologies, referring to Fig. 1B. To help the readers understand this schematic figure, we state additional details: (1) If the yellow loop is formed but the blue loop is not formed, then the gene is expressed. Moreover, gene expression can be enhanced; (2) If both the yellow loop and the blue loop are formed, then the gene is also expressed. However, the transcriptional rate may be different from that in the former case since experiment data^21^ suggested that the formation of one DNA loop modulate the transcriptional rate of another DNA loop. In addition, the expression effect may also be different between cross-type and inline-type structures. Specifically, for the former, the formation of the blue loop represses the effect that the yellow loop enhances gene expression, whereas for the latter, the case is just opposite; (3) If neither the yellow loop nor the blue loop is formed, then the gene is not expressed; (4) If the yellow loop is not formed but the blue loop is formed, then the gene is not expressed either.

Next, we map the three physical models in Fig. 1B into a common multistate model of gene expression, referring to Fig. 1C. With this mapping, a complex question of how two interacting DNA loops affects gene expression is transformed to a simple one of how a 4-state model of stochastic transcription is solved. Note that after mapping, the looping rates as functions of loop lengths currently become transition rates between promoter activity states. In addition, once two DNA loops are formed, any one of them can impact the length of the other, often in a nonlinear manner. Moreover, this impact can lead to changes in transition rates and further in the mRNA level. Also note that the ON1 and ON2 states indicated in Fig. 1C mean that the enhancer and promoter pair forms a loop (corresponding to the yellow loop) whereas the other pair of elements may form a loop (corresponding to the blue loop) but also may not form any loop. The transcriptional rates in ON1 and ON2 states may be different due to the interaction between two DNA loops. In contrast, at OFF1 and OFF2 states, the enhancer and the promoter pair does not form a loop whereas the other pair of regulatory elements may form a loop but also may not form any loop.

According to the above mapping relationships between physical and theoretical models, we know that the rates of loop dissociation and association, *λ*_12_, *λ*_21_, *λ*_23_, *λ*_32_, *λ*_43_, *λ*_34_, *λ*_14_, *λ*_41_ (unit: bp/second), are transformed to transition rates between active and inactive states shown in Fig. 1C. The former rates depend on the lengths or distances of the two loops (along the DNA lines), so do the latter rates. In the case of single DNA loops, previous works gave experiential formulae for the relationship between the looping rate (unit: bp/second) and the loop length (unit: *bp*)^19,36^. In our case, these formulae read

1 $$\begin{array}{c}{\lambda }_{14}={k}_{loop}^{(1)}=\beta \,\exp (-\frac{u}{{d}_{1}}-v\,\mathrm{log}({d}_{1})+w{d}_{1}+z)\\ {\lambda }_{{\rm{12}}}={k}_{loop}^{(2)}=\beta \,\exp (-\frac{u}{{d}_{2}}-v\,\mathrm{log}({d}_{2})+w{d}_{2}+z)\end{array}$$

where ${k}_{loop}^{(i)}$ represent the DNA looping rates, *d*_*i*_ are the loop lengths along the DNA lines, and *u*,*v*,*w*,*z* are parameters, the values of which can be obtained by fitting experimental datas^19,36^, e.g., *u* = 140.6, *v* = 2.52, *w* = 0.0014, and *z* = 19.9. Parameter *β* is a normalized constant for which we set *β* = 1/1000 throughout this article.

In general, each of two transition rates, *λ*_23_ and *λ*_43_ is a function of two distances, *d*_1_ and *d*_2_. However, the existing experimental datas support only the quantitative relationship between the DNA looping rate and the length of the yellow loop^14^. Based on the above analysis and without loss of generality, we may set *λ*_23_ = *k*_1_ *λ*_14_, *λ*_43_ = *k*_2_ *λ*_12_, where parameters *k*_1_ and *k*_2_ are set, based on experimental datas^14^ with small modifications, as

2a $${k}_{1}=\{\begin{array}{cc}0.73+\frac{1}{{d}_{1}} < 1 & {\rm{Alternating}}\,{\rm{loops}}\\ 40\,{e}^{-0.05{d}_{1}}+1 > 1 & {\rm{Nested}}\,{\rm{loops}}\\ 1 & \mathrm{Side}-\mathrm{by}-\mathrm{side}\,{\rm{loops}}\end{array}$$

2b $${k}_{2}=\{\begin{array}{cc}0.73+\frac{1}{{d}_{2}} < 1 & {\rm{Alternating}}\,{\rm{loops}}\\ 4 & {\rm{Nested}}\,{\rm{loops}}\\ 1 & \mathrm{Side}-\mathrm{by}-\mathrm{side}\,{\rm{loops}}\end{array}$$

Finally, we point out the following points: (1) DNA loops exist extensively, especially in ukaryotes^11–14^; (2) there are three representative types of interactions between DNA loops (alternating, nested and side-by-side loops), each supported by experimental data^11^; (3) there are two mainstream ways between DNA regulatory elements (direct looping and facilitated tracking looping), each supported also by experimental evidences^9,15,20^; (4) dependence of looping rates on loop lengths is obtained by fitting experimental data^19,36^; (5) our gene models that consider the interactions between DNA loops do not explicitly consider the regulatory roles of transcription factors (TFs), but if we let model parameters such as looping rates (or transition rates), and transcriptional rates change in their respective yet biologically reasonable ranges, then our models do not lost generality and in particular, they can capture the regulatory effects of TFs. This simplification, which has been adopted in many references^40–42^, is here made for analysis convenience. Moreover, one will see that the results obtained in this paper are qualitatively unchanged, independent of the choice of parameter vlues, so the simplification is reasonable.

### An approximate method for calculating mRNA probability distribution

One will see that energetic cost for the system under consideration has a closed relation with the probability distribution of the mRNA molecule number. In order to calculate this distribution, here we propose a simple and intuitive yet effective method, which is based on the isothermal decomposition of probability. Let *x*_1_, *x*_2_, *x*_3_ and *x*_4_ represent the DNA proportions (or fractions) at states OFF1, OFF2, ON2 and ON1, respectively; *y* represent the mRNA concentration. Denote by *μ*_1_ and *μ*_2_ the mRNA synthesis rates at ON1 and ON2 states respectively (unit: *μM*/sec); and by *δ* the mRNA degradation rate (unit: *μM*/sec). The deterministic equations for the full reaction system take the form

3 $$\begin{array}{rcl}\frac{d{x}_{1}}{dt} & = & -({\lambda }_{12}+{\lambda }_{14}){x}_{1}+{\lambda }_{21}{x}_{2}+{\lambda }_{41}{x}_{4}\\ \frac{d{x}_{2}}{dt} & = & -({\lambda }_{21}+{\lambda }_{23}){x}_{2}+{\lambda }_{12}{x}_{1}+{\lambda }_{32}{x}_{3}\\ \frac{d{x}_{3}}{dt} & = & -({\lambda }_{32}+{\lambda }_{34}){x}_{3}+{\lambda }_{23}{x}_{2}+{\lambda }_{43}{x}_{4}\\ \frac{d{x}_{4}}{dt} & = & -({\lambda }_{43}+{\lambda }_{41}){x}_{4}+{\lambda }_{34}{x}_{3}+{\lambda }_{14}{x}_{1}\\ \frac{dy}{dt} & = & {\mu }_{1}{x}_{4}+{\mu }_{2}{x}_{3}-\delta y\end{array}$$

where *x*_1_ + *x*_2_ + *x*_3_ + *x*_4_ = 1, Solving Eq. (3) at steady state, we obtain the following expressions

4 $${x}_{1}^{s}=\frac{A}{E},{x}_{2}^{s}=\frac{B}{E},{x}_{3}^{s}=\frac{C}{E},{x}_{4}^{s}=\frac{D}{E},{y}^{s}=\frac{{\mu }_{1}D+{\mu }_{2}C}{\delta E}$$

with *A* = *λ*_21_ *λ*_32_(*λ*_41_ + *λ*_43_) + *λ*_34_ *λ*_41_(*λ*_21_ + *λ*_23_), *C* = *λ*_12_ *λ*_23_(*λ*_41_ + *λ*_43_) + *λ*_14_ *λ*_43_(*λ*_21_ + *λ*_23_), *B* = *λ*_12_ *λ*_32_(*λ*_41_ + *λ*_43_) + *λ*_12_ *λ*_34_ *λ*_41_ + *λ*_14_ *λ*_32_ *λ*_43_, *D* = *λ*_14_ *λ*_21_ *λ*_32_ + *λ*_12_ *λ*_23_ *λ*_34_ + *λ*_14_ *λ*_34_(*λ*_21_ + *λ*_23_), *E* = *A* + *B* + *C* + *D*, which are all the implicit functions of DNA loop lengths along the underlying DNA lines.

In order to derive the mRNA probability distribution in an intuitive manner, we consider 4 extreme cases. The time scale of DNA looping is in general slower compared to that of transcription, so if the gene is only at OFF1 state, then the mRNA always degrades without production, implying that the mRNA concentration follows an exponential distribution. Specifically, if we denote by *P*_1_(*y*) the mRNA distribution in this case, then *P*_1_(*y*) = (*A*)/(*E*)*δe* ^−*δy*^, where *A/E* is a weight. Similarly, the mRNA distribution only at OFF2 state, *P*_2_(*y*), is given by *P*_2_(*y*) = (*B*)/(*E*)*δe* ^−*δy*^. If the gene is only at ON2 state, then the mRNA is both produced and degraded, implying that the mRNA concentration, denoted by *P*_3_(*y*), follows a Poisson distribution. From a mathematical view, Poisson distribution and normal distribution can be apprixmated to each other, implying that ${P}_{3}(y)=\frac{C}{E}\frac{1}{\sqrt{2\pi }{\sigma }_{1}}\exp [-\frac{{(y-{{\rm{\Lambda }}}_{1})}^{2}}{2{{\sigma }_{1}}^{2}}]$, where ${{\rm{\Lambda }}}_{1}=\frac{({\mu }_{1})}{(\delta )}$ and ${\sigma }_{{\rm{1}}}^{{\rm{2}}}=\frac{{\mu }_{1}}{\delta }$, *C*/*E* is a weight. Similarly, the mRNA concentration only at ON1 state follows a normal distribution with a weight, i.e., ${P}_{4}(y)=\frac{C}{E}\frac{1}{\sqrt{2\pi }{\sigma }_{2}}\exp [-\frac{{(y-{{\rm{\Lambda }}}_{2})}^{2}}{2{{\sigma }_{2}}^{2}}]$, where ${{\rm{\Lambda }}}_{2}=\frac{({\mu }_{2})}{(\delta )}$ and ${\sigma }_{{\rm{2}}}^{{\rm{2}}}=\frac{{\mu }_{2}}{\delta }$. Since the gene must be and is only at one of 4 states, the total protein distribution at steady state, denoted by *P*(*y*), should be equal to the sum of the above 4 fractorial distributions, that is, *P*_1_(*y*) = *P*_1_(*y*) + *P*_2_(*y*) + *P*_3_(*y*) + *P*_4_(*y*). Thus, we obtain the following analytical probability density of the mRNA concertration at steady state

5 $$\begin{array}{rcl}P(y) & = & \frac{A}{E}\delta {e}^{-\delta y}+\frac{B}{E}\delta {e}^{-\delta y}+\frac{C}{E}\frac{1}{\sqrt{2\pi }{\sigma }_{1}}\exp [-\frac{{(y-{{\rm{\Lambda }}}_{{\rm{1}}})}^{2}}{2{\sigma }_{1}^{2}}]\\  &  & +\,\frac{D}{E}\frac{1}{\sqrt{2\pi }{\sigma }_{2}}\exp [-\frac{{(y-{{\rm{\Lambda }}}_{2})}^{2}}{2{\sigma }_{2}^{2}}]\end{array}$$

**Figure 2:**
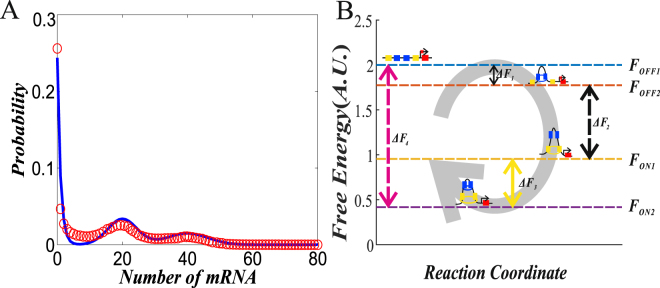
(**A**) Comparison probability distributions between analytical (solid line) and numerical (empty circles) results, where parameter values are set as λ_12_ = 0.4, λ_21_ = 0.2 λ_23_ = 0.2 λ_32_ = 0.3, λ_34_ = 0.1, λ_43_ = 0.1, λ_41_ = 0.5, λ_14_ = 0.5, *μ*_1_ = 20 *μ*_2_ = 40 and *δ* = 1. (**B**) Free energies at 4 states of nested loops structure, where the free energy of the OFF1 state is set as 2 and energy differences between the states (denoted by ${\rm{\Delta }}{F}_{i}$, $i=1,2,3,4$) are indicated. Parameter values are set as *d*_1_ = 500, *d*_2_ = 300 and λ_12_ = λ_32_ = λ_34_ = λ_41_ = 0.3.

This explicit expression is in good accordance with the one obtained by the Gillespie stochastic simulation algorithm^43,44^ (referring to Fig. 2A, where we have used the fact that the size of the probability distribution *P*(*x*) at *x* = *i* is equal to that of the area bounded by the corresponding probability density curve and the interval [*i* − 1/2, *i* + 1/2]), implying that the above approximation is effective. In other words, the total probability density is equal to the sum of the individual probability densities at disctete states.

### Quantifying the free energy cost for the formation of DNA loops

One main aim of this paper is to clearly show how the interaction between DNA loops results in the differences between free energies at different states. For this, we transform this issue into that of the free energy dissipation defined as the entropy production rate. For clarity, we first consider a simple sub-block of two states OFF1 and OFF2 in Fig. 1. Denote by *F*_1_ and *F*_2_ the free energies that the gene is at OFF1 and OFF2 states, respectively. Then, according to Ref. [31], we know that the ratio between two transition rates, *λ*_12_/*λ*_21_, is proportional to ${e}^{-\beta {\rm{\Delta }}{F}_{1}}$, i.e., ${\lambda }_{12}/{\lambda }_{21}\propto {e}^{-\beta {\rm{\Delta }}{F}_{1}}$, where Δ*F*_1_ = *F*_2_ − *F*_1_ represents the difference between free energies, *F*_1_ and *F*_2_ (i.e., the change in free energy), and *β* = 1/(*k*_*B*_ *T*) is a composite parameter of the Boltzmann constant and temperature (without loss of generality, we set *β* = 1 in our analysis). We can show that the free energy consumption for the OFF1–OFF2 block is given by ${\dot{\omega }}_{1}=\frac{d}{dt}\,{\omega }_{1}=J\,\mathrm{ln}\,\frac{{\lambda }_{12}}{{\lambda }_{21}}$, where *J* is a constant, and will be specified later. Thus, ${\dot{\omega }}_{1}={h}_{1}-J{\rm{\Delta }}{F}_{1}$, where *h*_1_ is also a constant. In other words, the relation between ${\dot{\omega }}_{1}$ and Δ*F*_1_ is linear, and the difference between the free energy consumption rate and the free energy difference is determined by a constant factor *h*_1_ and a constant multiplier *J*, where the former constant is interest of this paper whereas the latter constant depends usually on the hydrolyzes of ATPs (energetic molecules)^45–48^. Similarly, if we denote by *F*_3_ and *F*_4_ the free energies that the gene is at ON1 and ON2 states respectively, and by Δ*F*_2_ = *F*_3_ − *F*_2_, Δ*F*_3_ = *F*_4_ − *F*_3_ and Δ*F*_4_ = *F*_1_ − *F*_4_ the differences between free energies of the system’s states, then we have $\frac{{\lambda }_{23}}{{\lambda }_{32}}\propto {e}^{-\beta {\rm{\Delta }}{F}_{2}}$, $\frac{{\lambda }_{34}}{{\lambda }_{43}}\propto {e}^{-\beta {\rm{\Delta }}{F}_{3}}$ and $\frac{{\lambda }_{41}}{{\lambda }_{14}}\propto {e}^{-\beta {\rm{\Delta }}{F}_{4}}$. To help the reader’ understanding, we take the nested loops structure as an example to show the changes of free energy in each state, referring to Fig. 2B. For this figure, we give interpretations below. Since tree energy is a measuring index in statistic physics, and its size is unknown in most cases. Without loss of generality, we may assume that the free energy of the OFF1 state is 2 (due to the setting of *β* = 1). By calculating the free energy difference between different states, one can judge the energy cost of switching between these states. From Fig. 2B, we observe that increasing the number of promoter states can reduce free energy, implying that the formation of DNA loop needs free energy.

Furthermore, the energy dissipation rates, ${\dot{\omega }}_{i}$, can also be expressed using the differences between free energies, that is, ${\dot{\omega }}_{i}={h}_{i}-J\Delta {F}_{i}$, where *i* = 1, 2, 3, 4.

Finally, we set Δ*F* = Δ*F*_1_ + Δ*F*_2_ + Δ*F*_3_ + Δ*F*_4_, which represents the change in free energy for the cyclic promoter of the gene shown in Fig. 1C or D. Note that $\frac{{\lambda }_{12}{\lambda }_{23}{\lambda }_{34}{\lambda }_{41}}{{\lambda }_{43}{\lambda }_{32}{\lambda }_{21}{\lambda }_{14}}\propto {e}^{-\beta {\rm{\Delta }}F}$ or $\mathrm{ln}(\frac{{\lambda }_{12}{\lambda }_{23}{\lambda }_{34}{\lambda }_{41}}{{\lambda }_{43}{\lambda }_{32}{\lambda }_{21}{\lambda }_{14}})=h-\beta {\rm{\Delta }}F$, where *h* is a constant depending on both the Boltzmann constant and temperature. Thus, we obtain the following expression for the relationship between the dissipation rate of free energy for the gene promoter and the difference between the corresponding free energies

6 $$\dot{W}=h-J{\rm{\Delta }}F$$

This establishes the linear relationship between the free energy difference Δ*F* and the free energy dissipation rate $\dot{W}$. Recall that the size of the difference between free energies relies on hydrolyzes of energetic molecules such as ATPs^45–48^. Therefore, studying the free energy consumption rate in a system of stochastic gene expression can help us understand the roles of regulation factors or processes such as the interaction between DNA loops in controlling the expression level. In this paper, we are more interested in mean free energy consumption rate (or simply “mean energy”), whch is defined as the ratio of the energy dissipation rate over the mean mRNA. According to the formula (6), we know that the mean free energy consumption rate is proportional to the free energy required per mRNA. We will apply the minimal energy principle to speculate the mechanism of the interaction between DNA loops.

### An effective method for calculating the free energy cost of the whole system

Based on the above results, here we provide an effective method for calculating the free energy cost of the entire gene expression system. First, we introduce 4 logic variables, ${\tilde{x}}_{1}$, ${\tilde{x}}_{2}$, ${\tilde{x}}_{3}$ and ${\tilde{x}}_{4}$, to represent 4 promoter states, where every ${\tilde{x}}_{i}$ takes only 0 or 1, i.e., ${\tilde{x}}_{i}\in \{0,1\}$. Since the gene must be and is only at one of 4 states, we have the conservative condition: ${\tilde{x}}_{1}+{\tilde{x}}_{2}+{\tilde{x}}_{3}+{\tilde{x}}_{4}=1$. For a fixed set of ${\tilde{x}}_{1}$, ${\tilde{x}}_{2}$, ${\tilde{x}}_{3}$ and ${\tilde{x}}_{4}$, the Fokker-Plank equation for *y* (where *dy*/*dt* = *μ*_1_ *x*_4_ + *μ*_2_ *x*_3_ − *δy* ≡ *F*) can be approximated as

7 $$\frac{\partial P(y,t)}{\partial t}=-\frac{\partial }{\partial y}(F\,P-\frac{1}{2}\frac{\partial }{\partial y}({\rm{\Phi }}P))$$

where $${\rm{\Phi }}={\mu }_{1}{x}_{4}^{s}+{\mu }_{2}{x}_{3}^{s}+\delta {y}^{s}=\frac{{\mu }_{2}C+{\mu }_{1}D}{E}+\delta {y}^{s}=2\frac{{\mu }_{2}C+{\mu }_{1}D}{E}$$

According to refs^37,49–51^, we know that the dissipation rate of free energy can in general be expressed as

8 $$\dot{W}\equiv \frac{dW}{dt}=\sum _{A,B}({J}_{A\to B}-{J}_{B\to A})\,\mathrm{ln}\,\frac{{J}_{A\to B}}{{J}_{B\to A}}$$

where A and B represent the microscopic states of the underlying system, and *J*_*σ* → *σ*′_ represents the transition probability from state *σ* to state *σ*′. In our case, A and B represent the states specified by $A=({\tilde{x}}_{1},{\tilde{x}}_{2},{\tilde{x}}_{3},{\tilde{x}}_{4},y)$ and $B=({\tilde{x}}_{1},{\tilde{x}}_{2},{\tilde{x}}_{3},{\tilde{x}}_{4},y+{\rm{\Delta }}y)$, where the absolute |Δ*y*| is infinitesimal, and *y* is a continuous variable. Moroever, the following decomposition holds

9 $$\sum _{A,B}({J}_{A\to B}-{J}_{B\to A})\,\mathrm{ln}\,\frac{{J}_{A\to B}}{{J}_{B\to A}}=\sum _{{\tilde{x}}_{1},{\tilde{x}}_{2},{\tilde{x}}_{3},{\tilde{x}}_{4}}\int ({J}_{A\to B}-{J}_{B\to A})\,\mathrm{ln}\,\frac{{J}_{A\to B}}{{J}_{B\to A}}dy+\sum _{{\tilde{x}}_{1},{\tilde{x}}_{2},{\tilde{x}}_{3},{\tilde{x}}_{4}}{\dot{\omega }}_{y}$$

where the first term on the right-hand side of Eq. (9) represents the free energy dissipation along the hyperplane ${\tilde{x}}_{1}+{\tilde{x}}_{2}+{\tilde{x}}_{3}+{\tilde{x}}_{4}=1$ in the state space whereas the second term represents the free energy dissipation along the *y*-direction. Thus,

10 $$\dot{W}={\dot{W}}_{p}+{\dot{W}}_{y}$$

where

10a $${\dot{W}}_{p}=\sum _{{\tilde{x}}_{1},{\tilde{x}}_{2},{\tilde{x}}_{3},{\tilde{x}}_{4}}\int ({J}_{A\to B}-{J}_{B\to A})\,\mathrm{ln}\,\frac{{J}_{A\to B}}{{J}_{B\to A}}dy$$

10b $${\dot{W}}_{y}=\sum _{{\tilde{x}}_{1},{\tilde{x}}_{2},{\tilde{x}}_{3},{\tilde{x}}_{4}}{\dot{\omega }}_{y}$$

The left task is to specify the expressions of ${\dot{W}}_{p}$ and ${\dot{W}}_{y}$. In the Spporting Online Material of this paper, we have showed

11 $${\dot{W}}_{p}=\frac{{\lambda }_{12}{\lambda }_{23}{\lambda }_{34}{\lambda }_{41}-{\lambda }_{43}{\lambda }_{32}{\lambda }_{21}{\lambda }_{14}}{E}\,\mathrm{ln}\,\frac{{\lambda }_{12}{\lambda }_{23}{\lambda }_{34}{\lambda }_{41}}{{\lambda }_{43}{\lambda }_{32}{\lambda }_{21}{\lambda }_{14}}\equiv J\,\mathrm{ln}\,q$$

which is just the energy dissipation of a circulation per unit time (see refs^38,52,53^), where $J=\frac{{\lambda }_{12}{\lambda }_{23}{\lambda }_{34}{\lambda }_{41}-{\lambda }_{43}{\lambda }_{32}{\lambda }_{21}{\lambda }_{14}}{E}$ and $q=\frac{{\lambda }_{12}{\lambda }_{23}{\lambda }_{34}{\lambda }_{41}}{{\lambda }_{43}{\lambda }_{32}{\lambda }_{21}{\lambda }_{14}}$. Regarding to the calculation formula for ${\dot{W}}_{y}$, we can show (seeing the Spporting Online Material for details)

12 $$\begin{array}{rcl}\dot{W} & = & (\frac{{\lambda }_{12}{\lambda }_{23}{\lambda }_{34}{\lambda }_{41}-{\lambda }_{43}{\lambda }_{32}{\lambda }_{21}{\lambda }_{14}}{E})\,\mathrm{ln}\,\frac{{\lambda }_{12}{\lambda }_{23}{\lambda }_{34}{\lambda }_{41}}{{\lambda }_{43}{\lambda }_{32}{\lambda }_{21}{\lambda }_{14}}\\  &  & +\frac{2}{{\rm{\Phi }}}[\frac{2A+2B}{E}+\frac{{\delta }^{2}(C{\sigma }_{2}^{2}+D{\sigma }_{1}^{2})}{E}]+\frac{2\delta (A+B-C-D)}{E}\\  &  & +\frac{{\rm{\Phi }}}{2E}[(A+B){\delta }^{2}+\frac{C}{{\sigma }_{2}^{2}}+\frac{D}{{\sigma }_{1}^{2}}]\end{array}$$

## Results

### Influence of the DNA loop length on free energy consumption

**Figure 3:**
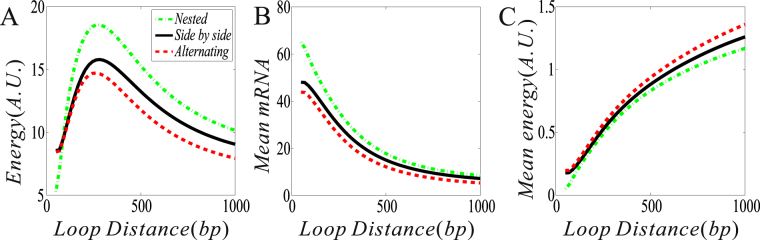
The effect of a DNA loop length on the free energy dissipation rate (energy) (no unit), the mRNA expression level, and the free energy dissipation rate(mean energy) (no unit). Where parameter values in all the cases are set μ_1_ = 40, μ_2_ = 80, *δ* = 1, γ = 0, *d*_2_ = 300, λ_12_ = λ_32_ = λ_34_ = λ_41_ = 0.3, and *d*_1_ ∈ (50, 1000).

As is well known, whether two DNA regulatory elements form a loop depends on the distance between them along the DNA line, and that this distance in turn can affect gene expression and further cell-to-cell variability^18,19^. Here, we investigate how DNA loop lengths in the possible structures of two interacting DNA loops impact the free energy dissipation rate (or simply “energy”, which is equal to the entropy production rate. See Methods for details), the mean mRNA, and the mean free energy consumption rate (i.e., “mean energy”). For clarity, we change a DNA loop length (*d*_1_) while keeping another DNA loop length (*d*_2_) fixed. The numerical results are shown in Fig. 3, where the value range of *d*_1_ is from the experimental datas (12).

From Fig. 3, we observe that for each of three fundamental structures (nested, side-by-side and alternating), there is an optimal loop length (*d*_1_) such that the energy dissipation rate reaches a maximum. Moreover, in contrast to the side-by-side structure, the nested structure has a larger energy dissipation rate (i.e., consuming more free energy) while the alternating structure has a smaller energy dissipation rate, referring to Fig. 3A. On the other hand, for each structure, the mean mRNA expression level is all a monotonically decreasing function of *d*_1_, but is higher for the side-by-side structure than for the alternating structure but lower than for the nested structure, referring to Fig. 3B. Although the energy dissipation rate is not a monotonic function of *d*_1_ (Fig. 1A), the mean energy dissipation rate is, referring to Fig. 3C. Interestingly, the order for three curves shown in Fig. 3A,B currently becomes opposite in the case of Fig. 3C (i.e., the case of mean energy consumption rate). Figure 3 indicates that in all the three structures, the nested structure produces most mRNAs and consumes lest mean free energy (meaning that generating one mRNA dissipates lest free energy). By contrast, the alternating structure produces fewest mRNAs and consumes most mean free energy (meaning that generating one mRNA dissipates most energy). Thus, we conclude that the nested structure performs best in all the three structures from the viewpoint of energy consumption.

Note that the further the distance between regulatory elements is, the more difficult they form a DNA loop or the smaller the DNA looping rate becomes. Thus, we also conclude that the faster the DNA looping becomes, the less mean free energy is dissipated and the more mean mRNAs is produced, whereas the slower the DNA looping becomes, the more mean free energy is consumed and the fewer mean mRNAs is produced.

### Influence of the interaction between DNA loops on free energy consumption

As is well known, the interaction between DNA loops would be complex^2,4,5,12,13^. Many questions, e.g., how the loop-loop interactions including communication forms affect gene expression and how DNA looping consumes free energy, remain elusive. In the last subsection, we have shown that if one DNA loop formation promotes another DNA loop formation (e.g., in the nested structure), then free energy can be saved (i.e., the promotion reduces free energy consumption). By contrast, if one DNA loop formation suppresses another DNA loop formation (e.g. in the alternating structure), then more free energy is consumed (i.e., the suppression increases free energy consumption). In this subsection, we consider another mode of the interaction between two DNA loops, i.e., one DNA loop is assumed to influence the transcriptional rate of another DNA loop.

To help the reader understand the results obtained in this subsection, let us recall our assumptions (see details in Material and Methods). The blue DNA loop formed by a pair of regulatory elements interacts with the yellow DNA loop formed by a pair of enhancer and promoter, and that the gene is not expressed in the former case but is expressed in the latter case. The former loop may affect the latter loop in two ways: promotion and suppression. Specifically, if the blue loop facilitates the formation of the yellow loop, i.e., if the former enlarges the looping rate of the latter or the transcription rate, then we call the corresponding case as promotion. If the blue loop reduces the looping rate of the yellow loop or the transcription rate, then we call the corresponding case as suppression. In the above subsection, we have analyzed the effect of looping rate on free energy consumption. In this subsection, we try to answer the question of how the promotion or the suppression impacts free energy consumption, mean mRNA expression and mean free energy dissipation. By numerical analysis, we find some universal phenomena (see the following contents). For clarity, we distinguish the following into two cases: one DNA loop promotes the other DNA loop (more precisely, the former enhances transcription of the latter); one DNA loop suppresses the other DNA loop. In the following, *μ*_1_ represents the transcription rate at ON1 (in this case, the yellow loop is formed but the blue loop is not formed, so *μ*_1_ may be understood as a fundamental transcription rate); *μ*_2_ represents the transcription rate at ON2 (in this case, both the yellow loop and the blue loop are formed, so *μ*_2_ may be understood as a regulated transcription rate). Note that *μ*_1_ < *μ*_2_ means that the blue loop formation promotes the yellow loop formation, whereas *μ*_1_ > *μ*_2_ means that the former prohibits the latter. Note that the size of *μ*_2_ can represent the interaction degree.

**Figure 4:**
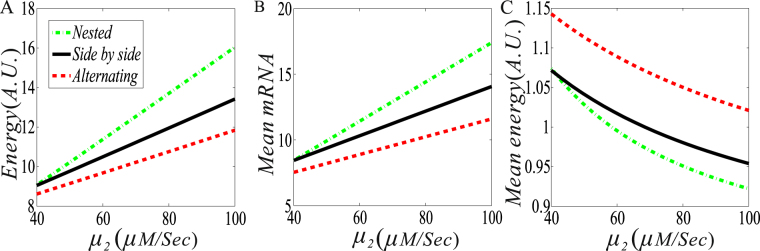
The effect of the promotion (i.e., ${\mu }_{1} < {\mu }_{2}$) of two DNA loops on free energy dissipation and mRNA expression level. (**A**) The dissipation rate of free energy vs the transcription rate (${\mu }_{2}$); (**B**) The mean mRNA expression level vs$\,{\mu }_{2}$; and (**C**) The mean free energy consumption vs ${\mu }_{2}$. In all the cases, parameter values are set as ${\mu }_{1}=40,\,{\mu }_{2}\in (40,\,100),\,{\rm{\delta }}=1,\,{\rm{\gamma }}=0,\,{d}_{2}=300,\,{d}_{1}=500,$ ${\lambda }_{12}={\lambda }_{32}={\lambda }_{34}={\lambda }_{41}=0.3$.

First, consider the case that one DNA loop enhances the expression of the other DNA loop, i.e., consider the case of *μ*_1_ *μ*_2_. From Fig. 4A, we observe that if the transcription rate *μ*_1_ is fixed, then the dissipation rate of free energy is a monotonically increasing function of the transcription rate *μ*_2_, whichever the structure of two DNA loops. However, there are differences in the amount of energy consumption among three fundamental structures. Specifically, in contrast to the side-by-side structure, the nested structure consumes more free energy due to the increase of the mRNA mean whereas the alternating structure consumes less free energy due to the decrease of the mRNA mean, independent of *μ*_2_ that must be larger than *μ*_1_ due to promotion. Results in the case of mean mRNA expression analysis is fundamentally the same as those in the case of free energy consumption rate analysis, referring to Fig. 4B. However, the change tendency and the order of three curves in the case of mean free energy dissipation are completely different from the case without making average, referring to Fig. 4C. Precisely, the mean free energy dissipation rate is a monotonically decreasing function of the transcription rate *μ*_2_, and the order of three curves is that the curve for the nested structure is below the curve for the side-by-side structure, which is below the curve for the alternating structure.

From Fig. 4, we can conclude some results as following:

1. Regardless of the structure of the interaction between two DNA loops, if *μ*_2_ increases (i.e., the promotion interaction is enhanced), then the mean RNA level will increase (following the increase in free energy consumption) and the mean energy consumption (i.e., the free energy consumed by the production of one mRNA) decreases. As such, we speculate that the promotion-type interaction may save free energy.
2. In contrast to the side-by-side structure, the nested structure consumes less mean free energy while the alternating structure consumes more free mean energy if one DNA loop facilitates the other DNA loop. The result is opposite otherwise.

**Figure 5:**
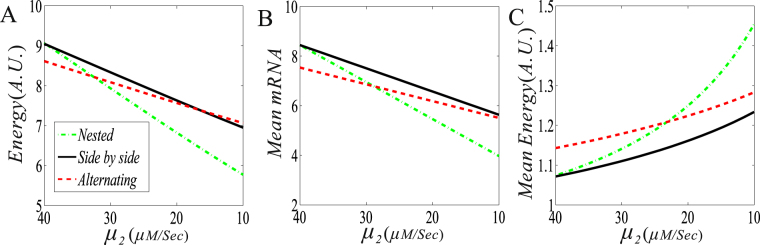
The effect of the suppression (i.e., ${\mu }_{1} > {\mu }_{2}$) of two DNA loops on free energy dissipation and mRNA expression level. (**A**) The dissipation rate of free energy vs the transcription rate (${\mu }_{2}$); (**B**) The mean mRNA expression level vs ${\mu }_{2}$; and (**C**) The mean free energy consumption vs ${\mu }_{2}$. In all the cases, except for ${\mu }_{2}\in (10,40)$, other parameter values are set as the same as in Fig. 4.

Then, consider the case that one DNA loop represses the expression of the other DNA loop, i.e., consider the case of *μ*_2_ < *μ*_1_. The numerical results are shown in Fig. 5. From Fig. 5A, we observe that in contrast to the side-by-side structure, both the nested and the alternating structures always consume less free energy since the corresponding curves are below the curve for the side-by-side loops. For a smaller *μ*_2_ of *μ*_2_ < *μ*_1_ (e.g., *μ*_2_ < 33 if *μ*_1_ = 40 is set), the nested structure consumes less free energy than the alternating structure, whereas for a larger *μ*_2_ of *μ*_2_ < *μ*_1_, the former consumes more free energy. The dependence of the mean mRNA level on *μ*_2_ has the similar change tendency to that of the free energy dissipation rate, but the critical value of *μ*_2_ for the cross point of two curves currently become smaller, referring to Fig. 5B. Then, we analyze Fig. 5C. We observe that the mean free energy consumption is a monotonically decreasing function of *μ*_2_, independent of the structure of two DNA loops. In contrast to the side-by-side structure, both the nested and the alternating structures consume more mean free energy since the corresponding curves are beyond the curve for the side-by-side loops. For a smaller *μ*_2_ of *μ*_2_ < *μ*_1_ (e.g., *μ*_2_ < 23 if *μ*_1_ = 40 is set), the nested structure consumes more free energy than the alternating structure, whereas for a larger *μ*_2_ of *μ*_2_ < *μ*_1_, the former consumes less mean free energy than the latter.

From Fig. 5, we can conclude that if the blue DNA loop suppresses the yellow DNA loop (meaning that the former reduces the transcription rate of the latter), then both the free energy dissipation and the mean mRNA level are reduced but the mean free energy consumption is increased, regardless of the structure between two interacting DNA loops. Moreover, the smaller the *μ*_2_ is (or the stronger the suppression is), such a reduction or increase becomes more apparently. Simply speaking, the suppression leads to the increasing of the mean free energy consumption (meaning that the production of one mRNA needs to consume more free energy). Therefore, we speculate that the suppression-type interaction needs to dissipate more free energy.

By comparing Figs 4 and 5, we obtain a universal conclusion, that is, the promotion of two loops dissipates more free energy but less mean free energy whereas the suppression consumes less free energy but more mean free energy, whichever the structure of two DNA loops.

### Influence of the communication form between regulatory elements on free energy consumption

In biology, a long-term debate is which of direct looping model and facilitated-tracking model is more reasonable. Here, we try to give an answer to this question from the viewpoint of free energy dissipation.

Before that, we first introduce a parameter to quantify the effect of the communication way between DNA regulatory elements on gene expression. Imagine a DNA loop as a string with a fixed length, two ends of which represent looping elements (e.g., a pair of Su and Hw, a pair of Anchor and CTCF). If one element slides along this string (here we only consider the sliding of one element in the blue loop), then this will affect the range that the enhancer and the promoter form the yellow loop. Thus, the tracking mechanism leads to the increase in looping rates. Specifically, if the looping rates of the yellow and blue loops are denoted by a pair of ${\tilde{\lambda }}_{14}$ and ${\tilde{\lambda }}_{{\rm{12}}}$ in the case that the facilitated-tracking mechanism exists, and their natural looping rates by another pair of *λ*_14_ and *λ*_12_, then the relationships between these pairs can be expressed as

13 $${\tilde{\lambda }}_{14}={\lambda }_{14}+{{\rm{\Delta }}}_{1},{\tilde{\lambda }}_{{\rm{12}}}={\lambda }_{{\rm{12}}}+{{\rm{\Delta }}}_{2}$$

where Δ_*i*_ represents the differences between the two cases. Similarly, a pair of *λ*_23_ and *λ*_34_ need to be modified. Note that for the facilitated-tracking mechanism, a longer DNA loop leads to a wider range for one regulatory element to track another regulatory element, implying Δ ∼ *d* or Δ = *rd*, where *r* is a nonnegative parameter. Thus, no tracking or direct looping corresponds to *r* = 0, whereas the facilitated-tracking model corresponds to *r* ≠ 0. In ref.^18^, the parameter *r* is called as the tracking ratio, which can be understood as the probability that the enhancer and the promoter track to each other along the DNA line.

**Figure 6:**
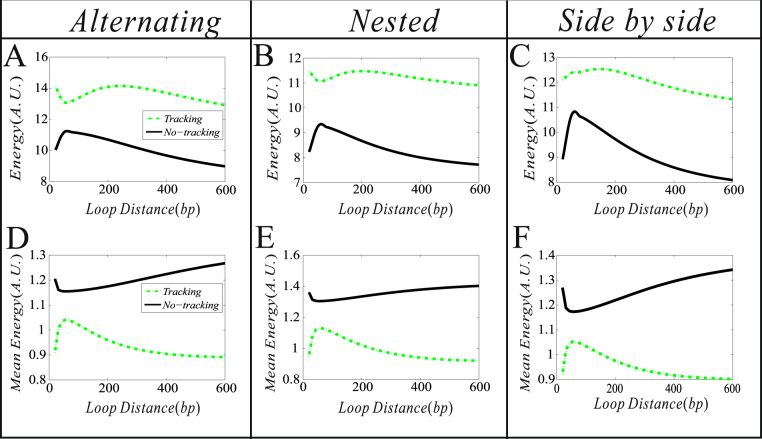
The dependences of energy dissipation rate and the mean energy dissipation rate on the length of the blue loop. Dashed lines corresponding to the facilitated-tracking model (${\rm{\gamma }}\ne 0$) whereas solid lines to the directing looping model (${\rm{\gamma }}=0$). In all the cases, parameter values are set as ${\mu }_{1}=40,{\mu }_{2}=80,{\rm{\delta }}=1,{\rm{\gamma }}=0.1,{d}_{2}\in (10,600),{d}_{1}=1000,$ ${\lambda }_{12}={\lambda }_{32}={\lambda }_{34}={\lambda }_{41}=0.3$.

Now, we examine the effect of the communication way between loop elements on free energy dissipation and on the mean free energy dissipation. First, investigate the dependences of free energy dissipation and the mean free energy dissipation on the length of the blue loop (*d*_2_) in both cases of tracking (corresponding to *r* ≠ 0) and of no tracking (corresponding to *r* = 0). The numerical results are shown in Fig. 6.

From Fig. 6, we first observe that more free energy is consumed in the case of tracking (e.g., *r* = 0.1) than in the case of no tracking (meaning *r* = 0) in all the three structures (comparing dash lines with solid lines in Fig. 6A–C). However, less mean free energy is dissipated in the case of tracking than in the case of no tracking (comparing dash lines with solid lines in Fig. 6D–F). This indicates that from the viewpoint of average, the facilitated-tracking mechanism is better in free energy dissipation than the direct looping mechanism. In addition, it implies that the former communication mechanism facilitates the mRNA expression than the latter communication mechanism since the mean free energy consumption is reduced. The above observation is the main result of this subsection.

**Figure 7:**
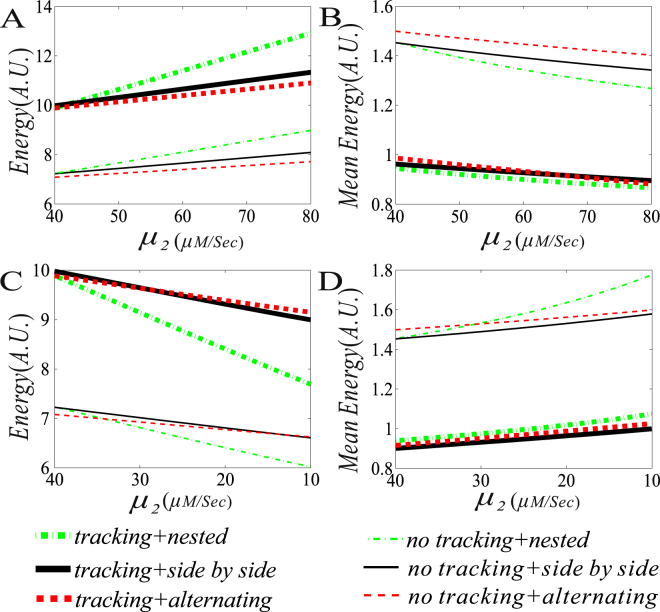
Comparison between the results for tracking and those for no tracking. (**A**) and (**B**), the free energy consumption rate/mean free energy consumption rate vs the transcription rate ${\mu }_{2}$, where the blue DNA loop is assumed to enhance the expression of the yellow DNA loop, ${\mu }_{1}=40$, and ${\mu }_{2}\in (40,80)$; (**C**) and (**D**), the free energy consumption rate/mean free energy consumption rate vs the transcription rate ${\mu }_{2}$, where the blue DNA loop is assumed to repress the expression of the yellow DNA loop, ${\mu }_{1}=40$, and ${\mu }_{2}\in (10,40)$. In all the cases, the other parameter values are set as ${\rm{\delta }}=1,{\rm{\gamma }}=0.1,{d}_{2}=600,\,{d}_{1}=1000$, and$\,{\lambda }_{12}={\lambda }_{32}={\lambda }_{34}={\lambda }_{41}=0.3$.

Next, we show the effects of promotion (i.e., *μ*_2_ > *μ*_1_) and suppression (i.e., *μ*_2_ < *μ*_1_) on free energy consumption and on its mean in the case that the facilitated-tracking mechanism is considered. For this, we compare the results in the case of tracking with those in the case of no tracking (i.e., direct looping), referring to Fig. 7. We observe from this figure that the more free energy is dissipated in the case of tracking (corresponding to thick curves in Fig. 7A,C) than in the case of no tracking (corresponding to thin curves in Fig. 7A,C). In contrast, the less mean free energy is dissipated in the case of tracking (corresponding to thick curves in Fig. 7B,D) than in the case of no tracking (corresponding to thin curves in Fig. 7B,D). These results are independent of the way of the interaction between two DNA loops.

**Figure 8:**
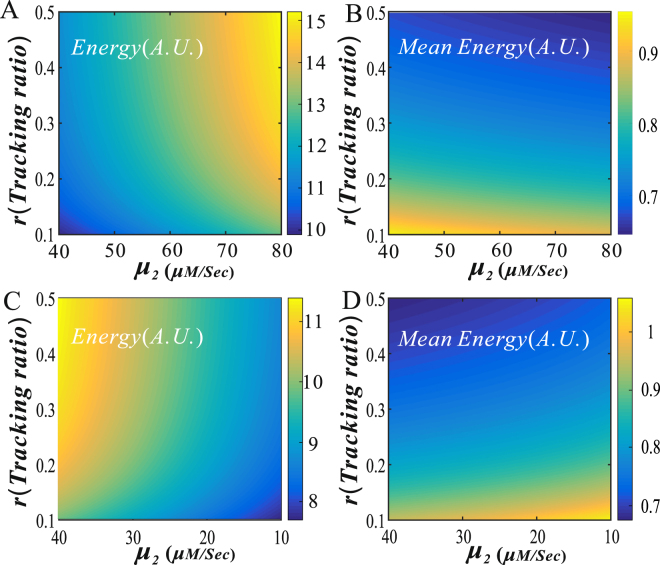
Three–dimensional pseudo diagrams for dependences of free energy dissipation rate/the mean free energy dissipation rate on both the transcriptional rate and the tracking ratio. (**A**) and (**B**) the blue loop promotes the transcription rate of the yellow loop, where parameters are set as ${\mu }_{1}=40$, ${\mu }_{2}\in (40,\,80)$, ${\rm{\delta }}=1,{\rm{\gamma }}\in (0.1,\,0.5),{d}_{2}=600,\,{d}_{1}=1000$, ${\lambda }_{12}={\lambda }_{32}={\lambda }_{34}={\lambda }_{41}=0.3$, ${k}_{1}=40\,{e}^{-0.05{d}_{1}}+1$,$\,{k}_{2}=4$, (**C**) and (**D**) the blue loop suppresses the transcription rate of the yellow loop, except for ${\mu }_{2}\in (10,40)$, other parameter values are set as the same as in (**A**).

In order to show the global effect of promotion (i.e., *μ*_1_ < *μ*_2_)/suppression (i.e., *μ*_1_ *μ*_2_) and the tracking ratio on both the free energy dissipation and the mean free energy dissipation, we further plot Fig. 8, a three–dimensional pseudo diagram. From the Figure, we clearly observe that the larger both the *μ*_2_ of *μ*_1_ < *μ*_2_ and the tracking ratio (*r*) are, the more is the total free energy is consumed (see Fig. 8A), but the less is the mean total free energy consumed (see Fig. 8B) since the mean mRNA level is increased. In contrast, the smaller the *μ*_2_ of *μ*_2_ < *μ*_1_ is and the tracking ratio (*r*) are, the less is the total free energy is consumed (see Fig. 8C), but the more is the mean total free energy consumed (see Fig. 8D) since the mean mRNA level is decreased. Thus, the results shown in Fig. 8 are in agreement with those shown in Fig. 7.

The above analysis indicates that the facilitated-tracking mechanism always reduces the mean free energy dissipation in contrast to the direct-looping mechanism. In addition, we have shown that the promotion-type interaction between two DNA loops may save free energy in contrast to the suppression-type interaction. Thus, according to the minimal energy principle, we speculate that the promotion-type interaction between two DNA loops plus the facilitated-tracking looping mechanism is the most possible way utilized by live organisms. Related biological reasons for this speculation are stated as follow.

First, two mainstream communication forms between DNA regulatory elements: direct looping and facilitated-tracking looping, exist extensively in reaslitic biological systems. For example, experimental data or evidence support the mechanism of facilitated-tracking looping between enhancer and promoter^54^, whereas other experimental data or evidence support the mechanism of direct looping between enhancer and promoter^55–57^. Second, that live organisms adopt which of the two mechanisms is a long-term debate in biology^15^. Third, experiments found that one DNA loop may influencce the expression of another DNA loops^22,58^. However, how they influence each other is not only unclear but also difficult to measure by an experimental method. Here we apply the the minimal energy principle to give a positive answer to this issue, as stated above.

## Discussion

Non-equilibrium mechanisms play important roles in many biological processes ranging from the concentration gradients that cells establish both with their environments and within themselves to chaperone-assisted protein folding and to gene expression. These non-equilibrium processes are essential for life as Ahsendorf, *et al*., ever pointed out that “we are only at equilibrium when we are dead”^59^, From the viewpoint of thermodynamics, non-equilibrium processes necessarily consume energy^38,39^. For example, the formation of a single DNA loop consumes about 9 kcal/mol but the corresponding energetic cost can be overcompensated by the interaction energy with transcription factors of some type that maintain the loop^60^. From the perspective of information theory, the entropy production rate is precisely the amount of energy consumption^53^. There is no energy consumption for detailed-balance systems but there is energy consumption for non-equilibrium steady-state systems^52^. However, quantitative analysis of free energy dissipation in biological systems, in particular in those of gene expression regulation, is nontrivial due to complexity of the involved biochemical processes. In this paper, by analyzing the free-energy costs of DNA looping and the interaction between interacting DNA loops, we have found universal results, e.g., whichever the structure of two loops (nested, side-by-side or alternating), the promotion of one DNA loop to another DNA loop (including increasing the looping rate and the transcription rate) always consumes less mean free energy whereas the suppression has the just opposite effect. More interestingly, we have shown that in contrast to the mechanism of direct looping between regulatory elements, the facilitated-tracking mechanism consumes less mean free energy but can enhance the mean mRNA expression. This result justifies the facilitated-tracking hypothesis, a long-standing debate in biology.

We have analyzed the free-energy costs in three fundamental structures of DNA-looping interactions (alternating loops, nested loops, and side-by-side loops), but enhancers and promoters may be connected in a highly complex network of DNA-looping interactions^61–63^, remarkably in eukaryotic cells. Since, many questions, e.g., at which step during gene activation, various nucleoprotein complexes assemble at distant enhancers, and how these complexes then contribute to promoter accessibility, the preinitiation complex recruitment and/or assembly, and transcription initiation and elongation, have been unsolved, the mechanisms for the energetic cost of gene expression have not been completely elucidated. In addition, enhancers have been shown to have a role in the preinitiation complex recruitment at target promoters^64–66^, the removal of proteasome complexes at promoters^67^, the generation of intra-chromosomal loops between regulatory regions^68^, and the regulation of elongation^69,70^; Enhancers are also involved in the removal of repressive histone modifications^71–74^, suggesting that they also contribute to the delivery of enzymes that regulate histone modifications^75,76^. In a word, enhancers in eukaryotic genomes can be many hundreds of kilobases away from the promoter they regulate^76, 77,78^, and the intervening DNA can contain other promoters and other enhancers^61,79,80^. All these complex cases would greatly complicate the investigation of the energetic cost in gene expression, and it is needed to develop new models and computational methods. However, our model has plasticity in many aspects, e.g., it can easily incorporate three main factors: connection pattern, distance between regulatory elements and communication form, which altogether can characterize interactions between chromatin loops.

From the perspective of applications, our method would provide a paradigm for analyzing the free-energy cost in gene expression involving complex regulatory processes. First, according to our proposed map method, we can map the topologies for the interactions among arbitrary DNA loops into a multistep model of gene expression, where DNA loop lengths (along the DNA lines) and other rates quantifying elaborate processes such as tracking between regulatory elements, energy-dependent chromatin remodeling, are easily incorporated into transition rates between promoter states, as done in this paper. This mapping is a key for one to investigate the corresponding energetic cost. Then, recall that the Gibbs energy is defined as

14 $$W=-k\int P({\boldsymbol{x}};t)\,\mathrm{ln}\,P({\boldsymbol{x}};t)d{\boldsymbol{x}}$$

where *k* = *k*_*B*_ *T* with *k*_*B*_ being a Boltzmann constant and *T* being temperature, and the entropy production rate that quantifies energy dissipation is defined as $\dot{W}=dW/dt$. To calculate $\dot{W}$, it is required to know the joint probability distribution *P*(***x***; *t*). However, even if we know that the Fokker-Planck equation for the underlying biochemical system is given by

15 $$\frac{\partial P}{\partial t}=-\sum _{i=1}^{n+1}\frac{\partial }{\partial {x}_{i}}({F}_{i}\,P-\frac{1}{2}\sum _{j=1}^{n+1}\frac{\partial }{\partial {x}_{j}}({{\rm{\Phi }}}_{i}P))\equiv -\sum _{i=1}^{n+1}\frac{\partial {J}_{i}}{\partial {x}_{i}}$$

where *x*_*i*_ quantifies promoter state *i* (e.g., representing the proportion of the DNA number at this state divided by the total DNA number), *F*_*i*_ represents dynamics of state *i* subjected to noise with the intensity Φ_*i*_ (1 ≤ *i* ≤ *n*), and *y* ≡ *x*_*n *+ 1_ represents gene production (mRNA or protein), it is very difficult to derive the expression of $\dot{W}$. In fact, we can only perform the formal calculation in this case (as done in most of the existing references):

$$\begin{array}{rcl}\dot{W} & = & -k\int [\mathrm{ln}\,P({\boldsymbol{x}};t)+1]\frac{\partial P({\boldsymbol{x}};t)}{\partial t}d{\boldsymbol{x}}\\  & = & k\sum _{i=1}^{n+1}\int [\mathrm{ln}\,P({\boldsymbol{x}};t)+1]\frac{\partial {J}_{i}}{\partial {x}_{i}}d{\boldsymbol{x}}\\  & = & -k\sum _{i=1}^{n+1}\int \frac{{J}_{i}}{P}\frac{\partial P}{\partial {x}_{i}}d{\boldsymbol{x}}\\  & = & \frac{k}{2}\sum _{i=1}^{n+1}\sum _{j=1}^{n+1}\int \frac{1}{P}\frac{\partial P}{\partial {x}_{i}}\frac{\partial ({\Phi }_{i}P)}{\partial {x}_{j}}d{\boldsymbol{x}}-k\sum _{i=1}^{n+1}\int {F}_{i}\frac{\partial P}{\partial {x}_{i}}d{\boldsymbol{x}}\end{array}$$

In spite of this, finding the distribution *P*(***x***; *t*) is another key for one to investigate the energetic cost and in general difficult, in particular in the cases that many complex processes associated with gene expression are considered. In this paper, we have proposed a simple approach to find *P*(***x***; *t*), which is based on the particular structure mapped from a complex network for the interactions among chromatin loops as well as the probability’s sum rule for independent events.

Finally, it should be pointed out that regulation (including the formation of DNA loops and the interaction between loops) is classically approached with thermodynamic methods^36–39^. We have shown that our model can be expressed in energetic terms and constitute a generalization of these approaches by extending the promoter structure, the range of systems that can be represented (i.e., including energy consuming systems such as eukaryotic promoters), and the type of metrics that can predicted (i.e., including measures of dynamic and stochastic properties). The usual thermodynamic formulation of cooperative and competitive association/dissociation of transcription factors (TFs)^31^ is equivalent to assign a Gibbs free energy to each promoter state. For our system, it corresponds to a 4-vector ***G***_0_ in the standard condition (i.e., assume that all TFs have unit concentration. For arbitrary concentrations, ${\boldsymbol{G}}={{\boldsymbol{G}}}_{0}+k{\sum }_{f\notin s}\,\mathrm{ln}\,[f]$, where [*f*] represents the TF concentration, *s* the set of TFs that are bound to the promoter at a given moment, and *k* is a constant related to the Boltzmann factor. Note that our model does not consider the second term in the total ***G*** since it does not consider TF regulations). This representation allows one to predict equilibrium steady-states (by applying the Boltzmann factor) and has been widely used to investigate the mean aspects of prokaryotic regulation^36,81^. But it has the drawback to restrict the analysis to energetically-closed systems and, not carrying any kinetic information, it forbids any investigation of the stochastic aspects of gene expression. For this energetic formulation to be equivalent to the kinetic one, one has to consider an additional set of energy values, which however are difficult to access experimentally, namely the energy of the activation barrier for each reaction.

## Electronic supplementary material

Supporting Online Material for The free-energy cost of interaction between DNA loops
