# Supplementary material for: The free-energy cost of interaction between DNA loops
Source: Sci Rep. 2017 Oct 3;7:12610. doi: 10.1038/s41598-017-12765-x (PMC5626758; doi:10.1038/s41598-017-12765-x)
Supplement: Supplementary file 1 — Supporting Online Material for The free-energy cost of interaction between DNA loops [file 41598_2017_12765_MOESM1_ESM.pdf]

Supporting Online Material for

The free-energy cost of interaction between DNA loops

Lifang Huang[1,2], Peijiang Liu[2], Zhanjiang Yuan[3], Tianshou Zhou[3*] and Jianshe Yu[1*]

[1] Research Centre of Applied Mathematics, Guangzhou University, Guangzhou 510006, P.R. China

[2]School of Statistics and Mathematics, Guangdong University of Finance & Economics, Guangzhou 510275, P.R. China

[3]Guangdong Province Key Laboratory of Computational Science, School of Mathematics and Computational Science, Sun Yat-Sen University, Guangzhou 510275, P.R. China

Derivation of Eqs. (11) and (12) in the main text

First, note that according to stochastic thermodynamics[1,2,3-6], $\dot{W}_p$ in the main text can be expressed as

$$\begin{aligned}\dot{W}_p =& \left(\tilde{P}(1,0,0,0)\lambda_{12} \ln\frac{\lambda_{12}}{\lambda_{21}} + \tilde{P}(0,1,0,0)\lambda_{21} \ln\frac{\lambda_{21}}{\lambda_{12}} \right) \\ &+ \left(\tilde{P}(0,1,0,0)\lambda_{23} \ln\frac{\lambda_{23}}{\lambda_{32}} + \tilde{P}(0,0,1,0)\lambda_{32} \ln\frac{\lambda_{32}}{\lambda_{23}} \right) \\ &+ \left(\tilde{P}(0,0,1,0)\lambda_{34} \ln\frac{\lambda_{34}}{\lambda_{43}} + \tilde{P}(0,0,0,1)\lambda_{43} \ln\frac{\lambda_{43}}{\lambda_{34}} \right) \\ &+ \left(\tilde{P}(0,0,0,1)\lambda_{41} \ln\frac{\lambda_{41}}{\lambda_{14}} + \tilde{P}(1,0,0,0)\lambda_{14} \ln\frac{\lambda_{14}}{\lambda_{41}} \right)\end{aligned} \qquad \text{(S1)}$$

where $\tilde{P}(1,0,0,0)=\frac{A}{E}$, $\tilde{P}(0,1,0,0)=\frac{B}{E}$, $\tilde{P}(0,0,1,0)=\frac{C}{E}$, $\tilde{P}(0,0,0,1)=\frac{D}{E}$, which represent the factorial probabilities that the gene is at states OFF1, OFF2, ON1 and ON2, respectively. Thus, we obtain Eq. (11) in the main text.

Then, we calculate $\dot{W}_y$ in the main text. In the case that $|\Delta y|$ is infinitesimal, we have approximation:

$$\frac{\partial}{\partial y}(\Phi P) \approx \frac{(\Phi P)(y+\Delta y)-(\Phi P)(y)}{\Delta y} \qquad \text{(S2)}$$

Using this approximation, the Fokker-Planck equation, i.e., Eq. (7) in the main text, becomes

$$\frac{\partial P(y,t)}{\partial t}=-\frac{\partial}{\partial y}\left[FP+\frac{1}{2}\frac{(\Phi P)(y)}{\Delta y}-\frac{1}{2}\frac{(\Phi P)(y+\Delta y)}{\Delta y}\right] \tag{S3}$$

If we denote $A'=\left(\tilde{x}_1^c,\tilde{x}_2^c,\tilde{x}_3^c,\tilde{x}_4^c,y\right)$ and $B'=\left(\tilde{x}_1^c,\tilde{x}_2^c,\tilde{x}_3^c,\tilde{x}_4^c,y+\Delta y\right)$, where every $\tilde{x}_i^c$ represents that the corresponding x_i is fixed, then along the y-direction in the phase space, the above transition probabilities in an infinitesimal range can be expressed as

$$J_{A'\to B'}=\left[\frac{FP}{\Delta y}+\frac{1}{2}\frac{(\Phi P)(y)}{\Delta y^2}\right]\Delta y,\quad J_{B'\to A'}=\left[\frac{1}{2}\frac{(\Phi P)(y+\Delta y)}{\Delta y^2}\right]\Delta y \tag{S4}$$

Thus, in the case that $|\Delta y|$ is infinitesimal, the energy consumption rate along the y-direction in the phase space is given by (see Ref. [7]),

$$\dot{\omega}_y=\sum_{A',B'}\left(J_{A'\to B'}-J_{B'\to A'}\right)\ln\frac{J_{A'\to B'}}{J_{B'\to A'}} \tag{S5}$$

Note that for an infinitesimal $|\Delta y|$, we have the approximation

$$\begin{aligned}
&\sum_{A',B'}\left(J_{A'\to B'}-J_{B'\to A'}\right)\ln\frac{J_{A'\to B'}}{J_{B'\to A'}}\\
&=\sum_{A',B'}\left[\left(\frac{FP}{\Delta y}+\frac{1}{2}\frac{(\Phi P)(y)}{\Delta y^2}-\frac{1}{2}\frac{(\Phi P)(y+\Delta y)}{\Delta y^2}\right)\ln\frac{\frac{FP}{\Delta y}+\frac{1}{2}\frac{(\Phi P)(y)}{\Delta y^2}}{\frac{1}{2}\frac{(\Phi P)(y+\Delta y)}{\Delta y^2}}\right]\Delta y\\
&\approx\sum_{A',B'}\left(FP-\frac{1}{2}\frac{\partial}{\partial y}(\Phi P)\right)\frac{2FP-\partial(\Phi P)/\partial y}{(\Phi P)(y+\Delta y)}\Delta y
\end{aligned}$$

Thus, $\Delta y\to 0$ yields the following formula for calculating energy dissipation rate along the y-direction in the phase space

$$\dot{\omega}_y=\int\frac{2J^2}{\Phi P}dy \tag{S6}$$

where $J=FP-(1/2)\partial(\Phi P)/\partial y$. Using the expression of $P(y)$ given by Eq. (5) in the main text, we can obtain Eq. (12) in the main text.

References

1. Zhang, X.J., Qian, H. & Qian, M. Stochastic theory of nonequilibrium steady states and its applications part I. *Phys. Rep*. **510**, 1-86(2012).
2. Qian, H. Open-system nonequilibrium steady state: statistical thermodynamics, fluctuations, and chemical oscillations. *J. Phys. Chem. B.* **110**, 15063-15074(2006).
3. Lebowitz, J. & Spohn, H. A Gallavotti-Cohen-type symmetry in the large deviation functional for stochastic dynamics. *J. Stat. Phys*. **95**, 333-365(1999).
4. Ruelle, D. P. Extending the definition of entropy to nonequilibrium steady states. *Proc. Natl. Acad. Sci. USA* **100**, 3054-3058(2003).
5. Qian, H. Phosphorylation energy hypothesis:open chemical systems and their biological functions. *Annu. Rev. Phys. Chem.* **58**, 113-142(2007).
6. Ge, H. & Qian, H. Physical origins of entropy production, free energy dissipation, and their mathematical representations. *Phys. Rev. E.* **81**, 561-578(2010).
7. Lan, G., Sartori, P., Neumann, S., Sourjik, V. & Tu, Y. The energy-speed accuracy trade-off in sensory adaptation. *Nat. Phys*. **8**, 422-428(2012).
